# Incidence and risk factors of warfarin therapy complications in community hospitals, central and eastern regions, Thailand: a retrospective, multicenter, cohort study

**DOI:** 10.1186/s13104-023-06383-2

**Published:** 2023-06-13

**Authors:** Benyapha Sombat, Sarisa Tongkaew, Aticha Nilwaranon, Mathirut Mungthin, Kanlaya Jongcherdchootrakul, Teeraboon Lertwanichwattana

**Affiliations:** 1 Phramongkutklao College of Medicine, Bangkok, Thailand; 2 Department of Parasitology, Phramongkutklao college of medicine, Bangkok, Thailand; 3 Department of Military and Community Medicine, Phramongkutklao college of medicine, Bangkok, Thailand

**Keywords:** Warfarin, Major bleeding, Thromboembolism, INR

## Abstract

**Objectives:**

Warfarin, like many other anticoagulants, has been linked to an elevated risk of bleeding proportional to the amount of anticoagulation used. Not only was the incidence of bleeding raised by the dosage, but the subtherapeutic international normalized ratio (INR) was also associated with increased thrombotic events. This retrospective cohort and multi-center study evaluated the incidence and risk factors of warfarin therapy complications in community hospitals in Thailand’s central and eastern regions from 2016 to 2021.

**Results:**

Among 335 patients (683.90 person-year of follow-up), The incidence rate of warfarin complications was 4.91 events per 100 person-year. The independent factor associated with warfarin therapy complications was propranolol prescription (Adjusted RR: 2.29, 95%CI: 1.12–4.71). The secondary analysis was divided according to the outcome of the major bleeding and thromboembolic event. Major bleeding events, hypertension (Adjusted RR: 0.40, 95%CI: 0.17–0.95), amiodarone prescription (Adjusted RR: 5.11, 95%CI: 1.08–24.15), and propranolol prescription (Adjusted RR: 2.86, 95%CI: 1.19–6.83) were the independent risk factors. While in the major thrombotic event, non-steroidal anti-inflammatory drugs (NSAIDs) prescription was an independent factor (Adjusted RR: 10.65, 95%CI: 1.26–90.35).

**Supplementary Information:**

The online version contains supplementary material available at 10.1186/s13104-023-06383-2.

## Introduction

Warfarin is an oral anticoagulant drug that is used for the prevention and treatment of thromboembolism events; for example, deep vein thrombosis (DVT), pulmonary embolism (PE), atrial fibrillation (AF), prosthetic-heart valve, embolic stroke, and post-myocardial infarction (post-MI). While the new generation of anticoagulants, especially the non-vitamin K antagonist oral anticoagulants (NOACs), are currently recommended by international guidelines for their efficacy and safety [1, 2]. However, NOACs are still not cost-effective at current prices in resource-limited healthcare places, especially in the community hospitals of Thailand [3–5].

As previously stated, warfarin has a narrow therapeutic index; hence, warfarin dosages for each patient rely on various criteria, including comorbidity, compliance, food and drug interactions, and alcohol consumption status. Several factors affect warfarin therapy and can cause problems. Bleeding and thromboembolic events occurred when the therapeutic index of warfarin was excessively and inadequately dosed, respectively. Bleeding and substantial hemorrhage were serious warfarin complications, such as intracranial hemorrhage, gastrointestinal bleed, ophthalmic bleeding, and so on. Furthermore, as the INR falls below 2, the risk of thromboembolism increases considerably, particularly in patients with atrial fibrillation [6]. Those who were either under or excessively anticoagulated at the time of the incident had significantly higher mortality and morbidity than those with optimal anticoagulation control [7].

There were three classifications for major bleeding events: fatal, major, and minor. Initially, the fatal bleeding complication was the autopsy- or radiologically- or clinically obvious death owing to hemorrhage. Second, the major bleeding complications included intracranial bleeding, retroperitoneal bleeding, intraocular bleeding, spontaneous muscle hematoma associated with compartment syndrome, any invasive procedure to stop bleeding, and active bleeding from any orifice in conjunction with unstable vital status. Minor bleeding, on the other hand, referred to any other form of bleeding not categorized as such above [8]. Hence, a fluctuating INR is frequently associated with a higher mortality rate [9, 10].

The same terminology applies to the significant issues arising from using warfarin. Despite this, the therapy response and therapeutic index adjustments were not the same for people of different ethnicities. Various studies have been carried out to investigate the outcome of warfarin therapy complications as well as the association among variables [11–13]. Since Asian patients are more sensitive to warfarin anticoagulation and the risk of bleeding adverse events tends to increase dramatically with minor increments in INR, this study will be essential to them [14]. Unfortunately, there were few studies in Thailand.

In addition, there was a lack of research on the complications of warfarin therapy in rural settings. Unless community hospitals were responsible for these areas, secondary prevention was essential for preventing future complications. Because of a rise in the population’s need for medical care, healthcare systems in countries, especially Thailand, are being pushed to provide primary care in community hospitals. As the daily struggle of overpopulation in community hospitals became more and more pressing, it became increasingly important to determine how often INR levels should be measured. Thus, researchers aimed to determine the incidence and risk factors of warfarin therapy complications in community hospitals in Thailand’s central and eastern regions.

## Methods

This research was conducted in three second-level (F2) community hospitals in the provinces of Lopburi and Chachoengsao. Tha Luang and Tha Wung community hospitals were located in the province of Lopburi, while Bang Khla community hospital was located in the province of Chachoengsao. F2 hospitals are community hospitals with 30 to 90 beds staffed by general practitioners (GPs) who provide secondary care.

A retrospective, multicenter cohort study was conducted in patients who received a continuous course of warfarin for more than 6 months due to their underlying conditions in the community hospitals from 1st October 2016 to 30th September 2021. Any patient who had non-related warfarin complications and underlying diseases of bleeding disorders was excluded from the study. The baseline population (n = 335) was invited to the study to identify the incidence and risk factors of warfarin therapy complications. All the available information was received from the outpatient and inpatient department records. Data collected consisted of demographic characteristics (gender, age), weight, height, body mass index (BMI), smoking behavior, alcohol consumption, hospital level, comorbidities, warfarin-use information, and warfarin therapy complications such as major bleeding disorders (intracranial hemorrhage (ICH), gastrointestinal bleeding (GI bleeding), or bleeding which required a blood transfusion of more than 2 units) and thromboembolism events (ischemic stroke, PE, and DVT).

Using Asia-Pacific obesity recommendations [15], the operational definition of BMI was categorized as follows: underweight (< 18.50 kg/m2), normal weight (18.50–22.99 kg/m2), overweight (23.00–24.99 kg/m2), obese I (25.00–29.99 kg/m2), and obese II (≥ 30.00 kg/m2). For this study, smoking and alcohol consumption were categorized as never, former, and current. Never was a patient reported as having a current status variable value of “no” and zero number of variables. Patients identified as never but lacking number information were excluded. Former was defined as a patient whose current status was “no” and whose numerical value exceeded zero. Current status was defined as a patient who answered “yes” to the current status inquiry with a number value greater than zero or was absent. Additionally, the study centers were responsible for classifying the different types of hospitals. The Thai guidelines for the prescription of warfarin include preventing venous thrombosis, treating pulmonary embolism, mechanical prosthetic heart valves, valvular heart disease, and atrial fibrillation [16].

The complications of warfarin can be divided into 2 groups, major bleeding and major thrombotic events leading to hospitalization. Major bleeding was defined in our study by an inpatient stay with ICH, GI bleeding, and bleeding which needed a transfusion of more than 2 units of whole blood or packed red blood cells [17] according to the recommendations of the Scientific and Standardization Committee of the International Society of Thrombosis and Hemostasis. Another consecutive major thrombotic complications comprised ischemic stroke, PE and DVT [18]. Venous thromboembolism (VTE) was defined as objectively confirmed DVT or PE. Stroke was defined as the sudden onset of a distinct focal neurologic deficit in a location consistent with the territory of a major cerebral artery and conclusive imaging evidence. ICD 9 and 10 codes were used to identify patient diagnoses, whereas ATC codes were utilized to identify all medications. All events were internally vetted using the aforementioned criteria.

An incidence rate of 100 person-year of observation was calculated for each potential risk factor variable. The data were analyzed in two stages: first, representing a cohort of warfarin therapy complications in community hospitals, and second, major bleeding and thrombotic events as subgroups identified based on their electronic health records database.

The association between potential risk factors and the incidence of warfarin therapy complications was assessed using Pearson’s chi-square test for categorical variables. A 0.05 cut-off point was used for the *p*-value and applied in all statistical analysis. Crude relative risk (RR) and 95% confidence intervals (CI) were calculated for warfarin therapy complications and each of the other variables in bivariate analysis. Multivariate Poisson regression analysis was performed to obtain adjusted RR and 95% CIs. All analyses were conducted using STATA 16.0 for Windows.

The study protocol was approved by the Institutional Review Boards of the Royal Thai Army Medical Department (approval number: M001h/65). The medical and pharmacy information claimed data from the electronic healthcare records in community hospitals.

## Results

### Demographic characteristics

A total of 335 who had no history of warfarin therapy complications at the baseline of 1st October 2016 were enrolled in the study. Among registered participants, the average age was 68.99 ± 12.28 years (28 to 100 years). In all, 162 participants (48.36%) received continuous warfarin therapy in Tha Wung hospitals, 194 participants (57.91%) were female, and the average BMI was 25.02 ± 5.53 kg/m2. Additional participants’ baseline characteristics are presented in more detail [see Additional file 1]. The overall incidence rate of warfarin therapy complications was 4.91 events per 100 person-year.

### Risk factors for warfarin therapy complications

Univariate analysis with Poisson regression was used to analyze the data. The factors associated with statistically significant warfarin complications (P < 0.05) were being overweight (BMI ≥ 23.00 kg/m2) (Crude RR: 0.41, 95%CI: 0.20–0.85), and propranolol prescription (Crude RR: 2.37, 95%CI: 1.17–4.82) [see Additional file 2].

Multivariate Poisson regression analysis showed that propranolol prescription was independently associated with warfarin therapy complications. The participants with propranolol prescription had a 2.29 times higher risk of acquiring warfarin therapy complications (95% CI: 1.12–4.17) than those without propranolol prescription after adjusting for gender and age group. In contrast, others showed no significant association with gender. [see Additional file 3].

From the total number of patients, 22 (6.57%) and 12 (3.58%) were identified to have major bleeding events and major thrombotic events, respectively. This study was able to differentiate major bleeding events and major thrombotic events based on their demographic and behavioral profiles. There were 34 major complications, which were 22 events of major bleeding (GI bleeding 15 events, ICH 6 events, and need for blood transfusion 1 event) and 12 events of major thromboembolism (ischemic stroke 9 events, DVT 2 events, and PE 1 event), as summarized in Table 1.

**Table 1:** Incidence and characteristics of warfarin therapy complication divided by major bleeding and thrombotic events (N = 335)

Characteristics	No. enrolled	Major bleeding events	Major thrombotic events
		No. (%)	No. (%)
		N = 22 (6.57)	N = 12 (3.58)
**Age group (years)**			
Mean ± SD	68.99 ± 12.28	71.77 ± 10.06	75.75 ± 11.89
Min - Max	28–100	47–87	53–100
< 60	65 (19.40)	2 (3.08)	1 (1.54)
60–69	95 (28.36)	6 (6.32)	2 (2.11)
70–79	104 (31.04)	9 (8.65)	6 (5.77)
≥ 80	71 (21.19)	5 (7.04)	3 (4.23)
**Gender**			
Male	141 (42.09)	11 (7.80)	2 (1.42)
Female	194 (57.91)	11 (5.67)	10 (5.15)
**BMI (kg/m2)**			
Mean ± SD	25.02 ± 5.53	25.36 ± 5.90	23.20 ± 2.97
Min - Max	11.20–45.79	16.80–39.03	20.00–29.22
< 18.50	30 (9.38)	1 (3.33)	0 (0)
18.50–22.99	93 (29.06)	10 (10.75)	6 (6.45)
23.00–24.99	50 (15.63)	1 (2.00)	1 (2.00)
25.00-29.99	95 (29.69)	7 (7.37)	3 (3.16)
≥ 30.00	52 (16.25)	3 (5.77)	0 (0)
**Community hospitals**			
Tha Luang	162 (48.36)	12 (7.41)	7 (4.32)
Bang Khla	52 (15.52)	4 (7.69)	0 (0)
Tha Wung	121 (36.12)	6 (4.96)	5 (4.13)
**Smoking status**			
Non-smoker	298 (88.96)	20 (6.71)	12 (4.03)
Ex-smoker	27 (8.06)	1 (3.70)	0 (0)
Current smoker	10 (2.99)	1 (10)	0 (0)
**Alcohol drinking status**			
Non-alcohol drinking	303 (90.72)	21 (6.93)	12 (3.96)
Ex-alcohol drinking	19 (5.69)	1 (5.26)	0 (0)
Current alcohol drinking	12 (3.59)	0 (0)	0 (0)
**Indication Atrial fibrillation**			
No	131 (39.10)	6 (4.58)	3 (2.29)
Yes	204 (60.90)	16 (7.84)	9 (4.41)
**Indication Myocardial infarction**			
No	312 (93.13)	21 (6.73)	12 (3.85)
Yes	23 (6.87)	1 (4.35)	0 (0)
**Indication Cerebrovascular accident**			
No	288 (85.97)	20 (6.94)	9 (3.13)
Yes	47 (14.03)	2 (4.26)	3 (6.38)
**Indication Valvular heart disease**			
No	322 (96.12)	20 (6.21)	12 (3.73)
Yes	13 (3.88)	2 (15.38)	0 (0)
**Indication Deep vein thrombosis**			
No	316 (94.33)	21 (6.65)	12 (3.80)
Yes	19 (5.67)	1 (5.26)	0 (0)

SD; Standard deviation, BMI; body mass index, kg/m2; kilogram per square meter

** Overall incidence rate of warfarin therapy complications was 4.91 events per 100 person-year

### Risk factors for major bleeding and major thrombotic complications

Risk factor analysis for major bleeding complications was summarized in Table 2. The identified risk factors included hypertension (Adjusted RR: 0.40, 95%CI: 0.17–0.95), amiodarone prescription (Adjusted RR: 5.11, 95%CI: 1.08–24.15), and propranolol prescription (Adjusted RR: 2.86, 95%CI: 1.19–6.83). An additional univariate analysis of risk factors for major bleeding complications shows this in more detail [see Additional file 4]. While NSAIDs prescription was an independent factor of major thrombotic events (Adjusted RR: 10.65, 95%CI: 1.26–90.35) in Table 3. An additional univariate analysis of risk factors for major thrombotic events shows this in more detail [see Additional file 5].

**Table 2:** Univariate and Multivariate analysis of risk factors for major bleeding complications

Characteristics	No. of events	Person-years of follow-up	Incidence rate/100 person-years	Crude RR	95% CI	*p*-value	Adjusted RR	95% CI		*p*-value	
**Age group (years)**											
< 60	2	131.13	1.53	1.00							
60–69	6	186.66	3.21	2.50	0.41–1.17	0.378	2.75	0.51–14.68		0.237	
70–79	9	228.94	3.93	2.81	0.61–13.20	0.186	4.30	0.83–22.35		0.082	
≥ 80	5	125.19	3.99	2.29	0.44–11.80	0.322	3.08	0.50–19.00		0.225	
**Gender**											
Male	11	296.78	3.76	1.00							
Female	11	375.14	2.93	0.73	0.32–1.68	0.454	0.72	0.30–1.69		0.448	
**BMI (kg/m2)**											
18.50–22.99	10	177.18	5.64	1.00							
< 18.50	1	49.23	2.31	0.31	0.40–2.42	0.264	0.34	0.04–2.70		0.308	
23.00–24.99	1	112.26	0.90	0.19	0.20–1.45	0.109	0.24	0.03–1.93		0.181	
25.00-29.99	7	184.24	3.80	0.69	0.26–1.80	0.443	0.71	0.26–1.93		0.501	
≥ 30.00	3	120.73	2.48	0.54	0.15–1.95	0.344	0.68	0.17–2.72		0.586	
**Hypertension**											
No	11	178.75	6.15	1.00							
Yes	11	492.93	2.23	0.50	0.22–1.16	0.109	0.40	0.17–0.95		0.037	
**Amiodarone prescription**											
No	20	662.75	3.18	1.00							
Yes	2	9.17	21.81	4.69	1.10–2.50	0.037	5.11	1.08–24.15		0.039	
**Propranolol prescription**											
No	13	533.71	2.44	1.00							
Yes	9	138.21	6.51	2.88	1.23–6.73	0.015	2.86	1.19–6.83		0.018	

*Data were adjusted for age, gender, BMI, comorbidities and medication, RR; Relative risk, 95% CI; 95% confidence interval, BMI; body mass index, kg/m2; kilogram per square meter

**Table 3:** Univariate and Multivariate analysis of risk factors for major thrombotic complications

Characteristics	No. of events	Person-years of follow-up	Incidence rate/100 person-years	Crude RR	95% CI	*p*-value	Adjusted RR	95% CI	*p*-value	
**Age group (years)**	12	671.92	1.79	1.06	0.99–1.12	0.056	1.04	0.98–1.11	0.187	
**Gender**										
Male	2	296.78	0.67	1.00			1.00			
Female	10	375.14	2.67	3.63	0.80–16.59	0.096	2.55	0.53–12.15	0.241	
**BMI (kg/m2)**										
< 23	6	226.41	2.65	1.00			1.00			
≥ 23	4	417.23	0.96	0.42	0.12–1.48	0.175	0.96	0.84–1.10	0.592	
**NSAIDs prescription**										
No	11	662.05	1.66	1.00			1.00			
Yes	1	9.87	10.13	7.52	0.97–58.27	0.053	10.65	1.26–90.35	0.030	

*Data were adjusted for age, gender, BMI, and medication, RR; Relative risk, 95% CI; 95% confidence interval, BMI; body mass index, kg/m2; kilogram per square meter, NSAIDs; non-steroidal anti-inflammatory drugs

## Discussion

In this multicenter cohort study, we aim to examine the incidence and risk factors of warfarin-related complications in community hospitals. The incidence rate of problems associated with warfarin medication was 4.91 per 100 person-years. In the multivariate analysis of warfarin therapy complications, the participants who had a prescription for propranolol were considered an independently relevant factor. Hypertension, amiodarone prescription, and propranolol prescription were identified as independent risk factors for major bleeding complications during the subgroup analysis. However, the major bleeding events demonstrated that the prescription of NSAIDs was an independent factor.

Major bleeding included ICH [19, 20], GI bleeding that led to an inpatient admission [21], and bleeding with the transfusion of blood of more than 2 units [22, 23]. And thromboembolism events included ischemic stroke, PE, and DVT. Both events are serious complications of warfarin therapy. Several factors affected INR levels and led to the events. The results of this study have shown some related and different factors compared to other studies.

The incidence of warfarin therapy complication is 4.91 events per 100 person-year in the community hospitals study, which is higher than others in Thailand. The tertiary care hospital in the eastern part of Thailand conducted a prospective cohort study among 1604 patients with warfarin therapy and the incidence were 3.13 events per 100 person-year, but lower than other reports in the western population [24]. The overall incidence rate might be overestimated to some extent because the incidences of warfarin therapy complications increased in the older age group. However, most persons who did not participate in this study were adults who shortly received warfarin. Nevertheless, the findings regarding risk factors would not be affected because the reasons for not participating in the study were unlikely to be related to other demographic characteristics, underlying diseases, and warfarin therapy complications.

In the total population, the incidence of warfarin therapy complications significantly differed among groups of propranolol prescription. The propranolol users showed a higher incidence of warfarin therapy complications compared to those who were non-users. Using a multivariate Poisson regression model, we found that those who were propranolol users had approximately 2.30 times acquiring warfarin therapy complications. Then, subgroup analysis was performed, and propranolol prescription were identified as relevant to the incidence of major bleeding events. In the particular major bleeding event group, patients who were propranolol users had approximately 2.86 times acquiring major bleeding events. This could be explained by propranolol leading to increased INR levels in patients under warfarin therapy [25]. Propranolol is a nonselective b-blocker that has been used to treat healthy people, and prior research has shown that it can increase the plasma levels of warfarin in these patients [26–28]. Therefore, it has been hypothesized that beta blockers like propranolol, which impede the metabolism of coumarin anticoagulants, may reduce the amount of warfarin that anticoagulated patients need to take to maintain their therapeutic index.

In 1988, patients prescribed amiodarone had an abnormal range of therapeutic prothrombin time (PT), indicating warfarin therapy complications compared with those who did not take amiodarone [29]. Besides that, recent studies revealed that amiodarone was a strong hepatic and renal metabolism inhibitor through cytochrome P450 pathways, including CYP2C9 [15]. These pathways were the metabolisms of warfarin. After the metabolisms of warfarin were reduced, the renal clearance of warfarin levels declined, which adjusted the pharmacokinetics and complications [30]. In community hospitals, amiodarone was on the Thai National Drug Lists which had to be at the hospitals. Moreover, amiodarone is more likely to be used in treating palpitation due to its promptness in emergency conditions. This is a reason why physicians lacked concern in the prescription of this medication. The authors suggested physicians and pharmacists implement alarm systems for drug interactions in electronic prescribing systems.

Inconsistent evidence suggested that having high blood pressure lowered the chance of warfarin treatment complications. Yet, we believed that hypertension patients administered warfarin would be well observed. Clinicians will have control over the procedure as a result of blood pressure monitoring. Throughout this treatment, the follow-up visit did not just focus on general characteristics and vital status. Yet, it continued to adhere to laboratory evaluations. It was obvious that regular monitoring would reduce the incidence of diseases and complications as a result of enhanced patient awareness. Furthermore, those with more diseases contacted medical services more frequently. Then, as a result of being hypertensive, their blood pressure and medication will be tightly controlled in warfarin-treated patients [31].

In the subgroup analysis of major thrombotic events, this study mainly focused on the prothrombotic effect of NSAIDs in continuously received warfarin patients. There were four population-based studies of nonselective NSAIDs and the risk of ischemic stroke conducted in Denmark, the United Kingdom, the United States, and The Netherlands [32–35]. They discovered that for a number of nonselective NSAIDs, such as ibuprofen, indomethacin, diclofenac, and naproxen, the odds ratio for ischemic stroke ranged from 1.2 to 1.7. Moreover, this medication had a statistically significant elevated risk of deep vein thrombosis and pulmonary embolism, according to a meta-analysis (pooled risk ratio 1.80, 95% CI: 1.28 to 2.52) [36]. After comparing to this study, NSAIDs prescription had a chance of major thrombotic events complications 10.65 times compared to non-NSAIDs users, prompting the physicians to advise caution when prescribing this medication.

In conclusion, the warfarin therapy monitoring program in community hospitals should be promoted by the Ministry of Public Health. A strategic approach to the prevention of warfarin therapy complications program, should include the suitability of warfarin dosage and other factors which could affect the INR level. Health education for both primary physicians and patients that emphasizes a management and prevention program should be implemented.

## Limitations

This may have contributed to an underestimation of the population’s risk assessment. Furthermore, the study was limited in its ability to access specific information from secondary data, including information on nutrition, smoking, and drinking status. Second, this research was undertaken in two provinces that may not be representative of the country in its entirety. The community’s electronic health records could not be the explanation for the representation from other regions of the country.

## Electronic supplementary material

Below is the link to the electronic supplementary material.

Supplementary Material 1

Supplementary Material 2

Supplementary Material 3

Supplementary Material 4

Supplementary Material 5

## Data Availability

The data that support the findings of this study are available from Tha Wung, Tha Luang, and Bang Khla hospitals, but restrictions apply to the availability of these data, which were used under license for the current study, and so they are not publicly available. Data are, however, available from the authors upon reasonable request and with permission of Tha Wung, Tha Luang, and Bang Khla hospitals.

## List of abbreviations

95% Ci: 95% confidence intervals
AF: Atrial fibrillation
BMI: Body mass index
DVT: Deep vein thrombosis
GI bleeding: Gastrointestinal bleeding
ICH: Intracranial hemorrhage
INR: International normalized ratio
NCDs: Non-communicable diseases
NOACs: Non-vitamin K antagonist oral anticoagulants
NSAIDs: Non-steroidal anti-inflammatory drugs
PE: Pulmonary embolism
post-MI: Post-myocardial infarction
PT: Prothrombin time
RR: Relative risk
VTE: Venous thromboembolism
